# Prenatally traumatized mice reveal hippocampal methylation and expression changes of the stress-related genes *Crhr1* and *Fkbp5*

**DOI:** 10.1038/s41398-021-01293-y

**Published:** 2021-03-23

**Authors:** Anne-Christine Plank, Stefan Frey, Lukas Andreas Basedow, Jalal Solati, Fabio Canneva, Stephan von Hörsten, Oliver Kratz, Gunther H. Moll, Yulia Golub

**Affiliations:** 1 Department of Child and Adolescent Mental Health, University Hospital Erlangen, Schwabachanlage 6 and 10, 91054 Erlangen, Germany; 2 Department of Child and Adolescent Psychiatry, Faculty of Medicine, Technische Universität Dresden, 01307 Dresden, Germany; 3 Department Experimental Therapy, University Hospital Erlangen and Preclinical Experimental Animal Center, Friedrich-Alexander-University Erlangen-Nürnberg, Palmsanlage 5, 91054 Erlangen, Germany

**Keywords:** Epigenetics and behaviour, Psychiatric disorders, Molecular neuroscience

## Abstract

In our previous study, we found that prenatal trauma exposure leads to an anxiety phenotype in mouse pups, characterized by increased corticosterone levels and increased anxiety-like behavior. In order to understand the mechanisms by which aversive *in utero* experience leads to these long-lasting behavioral and neuroendocrine changes, we investigated stress reactivity of prenatally traumatized (PT) mice, as well as the expression and methylation levels of several key regulatory genes of the stress axis in the dorsal hippocampus (dHPC) of the PT embryo and adult mice. We detected increased corticotropin-releasing hormone receptor 1 (*Crhr1*) and decreased FK506 binding protein 5 (*Fkbp5*) mRNA levels in the left dHPC of adult PT mice. These alterations were accompanied by a decreased methylation status of the *Crhr1* promoter and an increased methylation status of the *Fkbp5* promoter, respectively. Interestingly, the changes in *Fkbp5* and *Crhr1* mRNA levels were not detected in the embryonic dHPC of PT mice. Together, our findings provide evidence that prenatal trauma has a long-term impact on stress axis function and anxiety phenotype associated with altered *Crhr1* and *Fkbp5* transcripts and promoter methylation.

## Introduction

The developmental triad of growth, maturation, and learning is known to be modulated by an individual´s early environment^1^. Accordingly, early life experiences, including exposure to the maternal environment, set the growth trajectory of the child and are recognized as a key factor for disease susceptibility later in life^2^. Developmental programming, defined as the persisting effects of *in utero* events on tissue structure and function, has been shown to impact psychological, behavioral, neuroendocrine, and cardio-metabolic processes in adulthood (see for review^3,4^). From an evolutionary point of view, developmental programming is an adaptation strategy, intended for fitting the individual organism into a prospected “future environment” and hence beneficial for survival. However, when there is a mismatch between early and later life environment, these changes — originally intended to be “beneficial” — all together or in parts suddenly become maladaptive^5^. Such specific and often trauma-associated maladaptation increases an individual’s disease susceptibility^4,5^. For instance, children of mothers who experienced a traumatic event during pregnancy are at higher risk to develop a psychiatric disorder later in life^6–8^.

In this context, a considerable body of clinical and preclinical research has focused on trauma-related functional changes of the hypothalamic–pituitary–adrenal (HPA) axis^9^. Activation of the HPA axis is driven by hypothalamic secretion of corticotropin-releasing hormone (CRH), triggering pituitary-derived ACTH release and ultimately adrenal secretion of cortisol (human)/corticosterone (CORT, rodents). CRH is a key factor in the stress response of the brain, and the CRH/CRHR1 (CRH receptor 1) system — along with FK506 binding protein 5 (FKBP5) — is known to be critical in mediating susceptibility to anxiety disorders^10^. In rodents, early life stress has been found to induce an increased anxiety-like behavior and cognitive deficits in association with increased central CRH and CRHR1 expression^11^. CRH secretion and stress signaling are regulated by a negative feedback loop, which is activated via cortisol binding to the glucocorticoid receptor (GR/NR3C1). The sensitivity of the GR, in turn, is modulated by FKBP5, whose dysregulation has previously been linked to PTSD and other anxiety disorders^12^. In contrast to the early assumption that lowered basal cortisol is a hallmark of PTSD, meta-analyses showed that this holds true only for subgroups of patients and depends on the type and the time point of the trauma^13,14^.

Maternal PTSD has been associated with changes in the HPA axis regulation of the offspring, implicating maternally derived glucocorticoid programming in the intergenerational transmission of trauma-related changes^6–8^. Due to its lipophilic nature, maternal cortisol can pass through the placenta and enter the fetal circulation. Thus, stress-induced high maternal cortisol concentrations can impair the fragile balance of the developing HPA axis of the fetus. Such an impact on the programming of the fetal cortisol responsiveness can permanently influence an individual’s reactivity to stressful stimuli during its lifetime^7^. The molecular mechanisms by which prenatal stimuli trigger neurodevelopmental alterations in crucial brain regions are likely to involve epigenetic changes, such as modifications in DNA methylation profiles, resulting in altered expression of target genes^9^.

In our previous work, we established a mouse model of prenatal trauma (PT) based on the application of an electric foot shock to C57BL/6N female mice on the gestational day (GD) 12 during their pregnancy^15–17^. We found high anxiety levels and poor maternal care along with reduced serum prolactin and increased CORT levels in dams following maternal trauma (MT). Moreover, prenatally traumatized pups (PT pups) were born smaller and stayed smaller throughout their life. Additionally, when raised by a traumatized mother, PT pups had higher serum CORT levels and displayed increased anxiety-like behavior^17^.

The present work was conducted to better understand the long-term effects of prenatal trauma and/or being raised by a traumatized mother on the regulation of the stress system. We investigated epigenetic changes in several key regulatory genes of the stress axis in the hippocampus of PT mice, including *Crhr1*, *Fkbp5, Nr3c1*, and *Nr3c2* (encoding the mineralocorticoid receptor). In order to distinguish prenatal vs. postnatal effects, we examined samples collected from experimental animals sacrificed at GD 18 and at postnatal day (PND) 150. We focused on changes in the hippocampus, since it is crucially involved in fear memory processes and the regulation of the CRH system^18,19^.

We hypothesized that: (1) PT mice would display increased basal and stress-induced CORT levels; (2) changes in the CORT levels would go along with changes in the expression and methylation levels of key HPA axis regulatory genes; and (3) changes in the expression levels of the HPA axis regulatory genes following PT could be detected in embryos following *in utero* trauma.

## Material and methods

### Animals

Adult male and female C57Bl/6NCrl mice were purchased from Charles River Germany GmbH (Sulzfeld, Germany) at the age of 8 weeks. Animals were housed under standard conditions with a 12:12-h light/dark cycle (lights on at 05:00 a.m.), controlled temperature (23 ± 1 °C) and with food and water *ad libitum*.

Breeding (1 female and 1 male per cage) was initiated at the age of 10 weeks after 2 weeks of acclimatization. GD 0 was counted on the 2nd day of pairing with the male, and putatively pregnant females were single housed and monitored daily to ascertain their condition. Pups were reared by their natural mothers, weaned on PND 25, and housed in groups of four with no more than two animals of the same litter. Experiments were performed with male offspring, and a maximum of two male mice per litter were included in a single experiment to avoid “litter effects”. Animals were randomly assigned to their respective group by a chance procedure. The experimenter was not blinded to the group status. Group sample size determination was based on respective power analyses performed for our previously published studies^15–17^. All experimental procedures were approved by the district governments of Middle Franconia (Regierung von Mittelfranken, Ansbach, Az 54-2532.1-32/10) and Lower Franconia (Regierung von Unterfranken, Würzburg, Az 55.2-2532-2-441), Bavaria, Germany, and performed in strict compliance with the European Union Directive for the care and use of laboratory animals (ARRIVE guidelines^20^).

### Experimental procedures

#### Trauma application

MT was induced by contextual fear conditioning (Multi-Conditioning System, TSE Systems, Bad Homburg, Germany) at GD 12 (corresponding to the second trimester of gestation in humans), as previously described^17^. Animals were placed into the shock chamber for application of an inescapable foot shock: following 198 s of habituation, a single electric foot shock (2 s, 1.5 mA) was delivered via the metal grid. Animals remained in the chamber for 60 s before being returned to their home cages. The control group (no MT) was exposed to the shock environment without an electrical shock delivery. After each trial, the fear conditioning boxes were cleaned thoroughly with 70% EtOH. The test was performed between 6 and 8 p.m., i.e., during the dark phase.

#### Elevated plus maze (EPM)

In order to measure stress reactivity, mice were exposed to a classical EPM paradigm, performed as previously described^17^. The EPM was composed of two open arms (30 × 5 cm), and two enclosed arms (30 × 5 × 15 cm) with an open roof and elevated to the height of 50 cm above the floor. Mice were placed individually in the center of the maze, facing one of the open arms and their behavioral performance was recorded for 5 min by a camera positioned 1.5 m above the center of the maze. The test was performed between 11 a.m. and 2 p.m. during the light phase.

### Experiments

#### Experiment 1: the impact of PT on basal and stress-induced CORT levels and on hippocampal expression and methylation levels of HPA axis key genes in adult mice

**Fig. 1:**
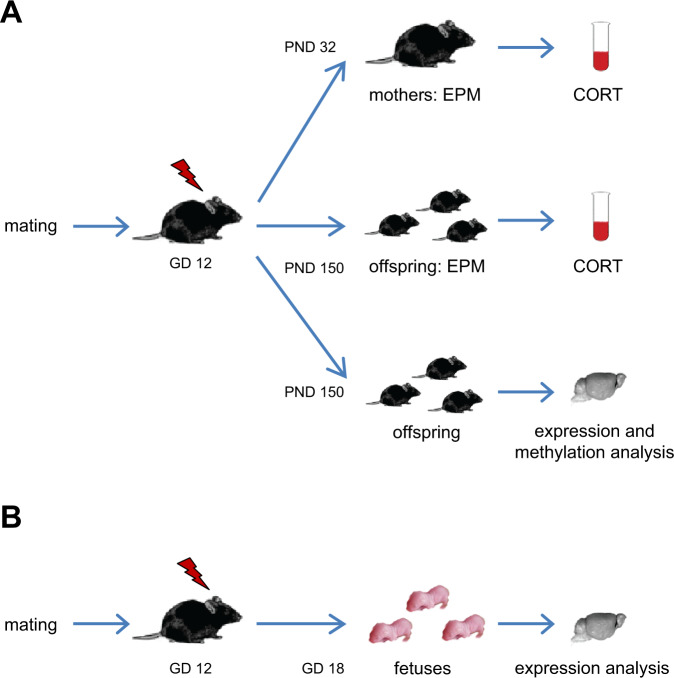
Experimental scheme. Schematic overview of experiment 1 (**A**) and experiment 2 (**B**). Lightning symbol = traumatic experience (electric foot shock) of the MT group; GD gestational day, PND postnatal day, EPM elevated plus maze.

Pregnant dams were subjected to trauma (MT; *n* = 14) or alternatively exposed to the shock chamber without application of the shock (no MT; *n* = 15), as described above (see Fig. 1A). Prenatally traumatized and control mice were subjected to further experimental procedures at the age of 21.5 weeks (PND 150), dams were tested 1 week after weaning (age: 18 weeks) (Fig. 1A):

- Dams were exposed to the mild stress of one EPM session for CORT measurements. Blood samples from each animal were taken at two different time points: basal (7 days before EPM session, immediately after separation from their pups) and post stress (60 min after behavioral testing). Blood was obtained from each animal (no MT: *n* = 13; MT: *n* = 12) via facial vein puncture (basal) and from the retro-orbital vein plexus (post stress) using hematocrit tubes under isoflurane narcosis. All sampling took place between 11 a.m. and 1 p.m. Serum preparation and CORT measurement were performed as described below.
- Similarly, basal (7 days before EPM session) and stress-induced (60 min after EPM session) serum CORT levels were measured in one group of PT male offspring (*n* = 10) and no PT control animals (*n* = 10). Blood was obtained from each animal via facial vein puncture (basal) and from the retro-orbital vein plexus (post stress) as described above.
- For gene expression and methylation analyses, tissue samples of PT and no PT mice (gene expression analyses: *n* = 7 per group; methylation analyses: *n* = 10 per group) were collected within 12 min after disturbing the cage. Animals were sacrificed via decapitation under short-term sedation with isoflurane (maximum exposure time: 10 s) and the brain was immediately isolated and shock frozen in liquid nitrogen. Frozen brains were coronally fixed and precut in a cryostat (CM3050S, Leica Biosystems, Wetzlar, Germany), followed by punching out a sample of the dorsal hippocampus (dHPC), which was defined based on a stereotaxic atlas (1.2 mm for dHPC, starting at 1.6 mm posterior to bregma). The dHPC samples were immediately frozen in liquid nitrogen and stored at −80 °C until further processing. The subsequent experimental procedures are described in detail below.

#### Experiment 2: the impact of prenatal trauma on embryonic expression levels of HPA axis key genes in the dHPC

In order to investigate whether gene expression changes in the dHPC occurred before birth, pregnant female mice (*n* = 7) were subjected to MT or exposed to the shock chamber without an application of the shock (no MT, *n* = 7) as described above (see Fig. 1B). On GD 18, 6 days after trauma exposure, pregnant mice were sacrificed and the fetuses isolated. Tissue sampling took place between 11 a.m. and 1 p.m. to ensure constant conditions and handling. Freshly isolated fetal brains were stored in RNAlater (Thermo Fisher, Waltham, MA, USA) for 24 h at 4 °C before dHPC isolation to prevent RNA degradation during the isolation process. Sampling of fetal dHPC tissue on GD 18 (no PT: *n* = 8; PT: *n* = 8) was performed as described in detail by Seibenhener and Wooten^21^. Hippocampal specimens were shock frozen in liquid nitrogen and stored at −80 °C until further processing.

### Corticosterone measurements

Blood was incubated for 45 min at room temperature to allow coagulation, and serum was subsequently separated at 2000 × *g* for 10 min, aliquoted and stored at −80 °C until the measurements were performed. CORT concentrations were determined using commercial ELISA kits (RE52211, IBL international GmbH, Hamburg, Germany) following the manufacturer’s instructions. All samples were assayed in duplicate using a microplate reader with corresponding software (Benchmark Plus™ microplate spectrophotometer, Bio-Rad Laboratories GmbH, Hercules, CA, USA) and quantified against a standard curve.

### RNA isolation and cDNA synthesis

RNA from dHPC specimens was extracted using the RNeasy Mini Kit (Qiagen, Hilden, Germany) according to the manufacturer’s instructions. Subsequently, RNA quality was determined by 260/230 and 260/280 ratios (Eppendorf Biophotometer, Eppendorf, Hamburg, Germany), as well as by gel electrophoresis. RNA samples, which fulfilled the MIQE guidelines^22^, were used for cDNA synthesis according to manufacturer’s protocol (Thermo Fisher, Waltham, MA, USA).

### qPCR

10 ng of cDNA was used per sample and gene for expression analyses of *Crhr1*, *Fkbp5, Nr3c1*, and *Nr3c2* by qPCR. Each sample was measured in triplicate using iQ™ SYBR^®^ Green Supermix (Bio-Rad Laboratories GmbH, Hercules, CA, USA) on a CFX95™ real-time cycler (Bio-Rad Laboratories GmbH, Hercules, CA, USA) running customized software. Cqs (Cycle at which the signal intersects the threshold between real signal and background) were determined by regression analysis and ΔCq values calculated on account of two reference genes (TATA box binding protein (*Tbp*) and beta-Actin (*Actb*)). According to quality standards, triplicates with a standard deviation ≥0.2 cycles were excluded. Outliers were identified using median ± 3* MAD for each sample. Expression folds were calculated using the 2^−ΔCq^ method and considering the respective primer efficiency. The following primer pairs were used:

*Crhr1*: fw: 5′-GCCCCATGATCCTGGTCCTGC-3′, rev: 5′-CCATCGCCGCCACCTCTTCC-3′;

*Fkbp5*: fw: 5′-GTGGGTTCTACATCGGCACT-3′, rev: 5′-GAGTCTGCGAAAGGACTTGG-3′;

*Nr3c1*: fw: 5′-AACTGGAATAGGTGCCA AGG-3′, rev: 5′-GAGGAGAACTCACATCTGGT-3′;

*Nr3c2*: fw: 5′-CATGGAGATTGTCAACGTCA-3′; rev: 5′-CTC GGCATCTCTCACAGAAT-3′;

*Tbp*: fw: 5′-CCTATCACTCCTGCCACACC-3′; rev: 5′-ATGACTGCAGCAAATCGC TTG-3′;

*Actb*: fw: 5′-GGCACCACACCTTCTACAATG-3′; rev: 5′-GGGGTGTTGAAGGTCTCAAAC-3′.

### Methylation analysis

Genomic DNA was isolated from dHPC samples using a commercial kit (QIAamp DNA Micro Kit, Qiagen, Hilden, Germany) following the manufacturer’s instructions and sent to Varionostic GmbH (Ulm, Germany) for EpiTYPER^®^ (Agena Bioscience, CA, USA) methylation analyses. Genomic DNA was treated with bisulfite to convert non-methylated cytosine to uracil, resulting in methylation-dependent sequence variations of C to T. These C/T variations appear as G/A changes on the reverse strand, which result in a mass difference of 16 Da per CpG site and can subsequently be detected by the MassARRAY system. The treated DNA was amplified by PCR and treated with shrimp alkaline phosphatase to neutralize unincorporated dNTPs. Subsequently, in vitro RNA transcription was performed, followed by base-specific cleavage at uracil residues using RNase A. The resulting fragments differ in mass and size, depending on the sequence changes due to bisulfite treatment, and generate characteristic signal patterns, which were identified by MALDI-TOF mass spectrometry.

### Data analysis and statistics

Data were analyzed using SPSS 21.0 (IBM, Armonk, NY, USA) and GraphPad Prism 8.0 (GraphPad Software Inc., San Diego, CA, USA). For CORT level analyses, normal distribution of data was confirmed by a Shapiro–Wilk test and a repeated measures two-way ANOVA (factors “MT”/“PT” and “pre/post stress”) was applied. For methylation analyses, two-tailed independent samples *t-*tests or *u-*tests, depending on a preceding test of normality (Shapiro–Wilk test), were used and *p* values were adjusted by false discovery rate procedure. Gene expression data were confirmed to be normally distributed (Shapiro–Wilk test) and analyzed via two-tailed independent samples *t-*test (fetal gene expression) or two-way ANOVA (factors “PT” and “hemisphere”) and post hoc Fisher’s LSD test. Similarity of variances between compared groups was confirmed by respective *F* tests. Values larger or smaller than the group mean ± 2 standard deviations were excluded from statistical analysis. Data are presented as mean values ± SEM. Statistical significance was accepted at *p* < 0.05.

## Results

### MT/PT affects basal and stress-induced CORT secretion

**Fig. 2:**
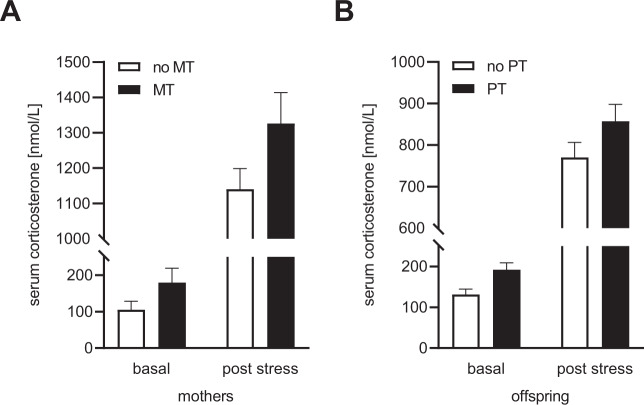
Serum CORT levels of MT and PT mice before and after stress exposure. **A** Basal CORT measurement of dams was performed 4 weeks after birth and 7 days before exposure to mild stress. MT dams showed increased basal and post stress CORT levels as compared to control animals. **B** In PT adult male mice, basal and post stress serum CORT levels were also higher than in no PT mice. See “Results” for detailed statistical analysis.

CORT measurements in dams 7 days before and 60 min after mild stress exposure revealed a significant effect of MT (*F*_1,23_ = 5.389, *p* = 0.03) and of stress exposure (*F*_1,23_ = 363.5, *p* < 0.0001), with increased CORT levels after mild stress exposure and elevated CORT levels in MT as compared to no MT mice (Fig. 2A). However, no interaction of both factors was revealed (*F*_1,23_ = 0.948, *p* = 0.34 [n.s.])

In their offspring, stress exposure also caused a significant increase in CORT levels, both in PT and no PT mice (*F*_1,18_ = 745.6, *p* < 0.0001) (Fig. 2B). Additionally, PT displayed higher CORT levels than no PT mice (*F*_1,18_ = 4.767, *p* = 0.043) (Fig. 2B). Again, no interaction effect was detected (*F*_1,18_ = 0.31, *p* = 0.584 [n.s.]).

### PT and/or being raised by a traumatized mother is associated with long-term changes in hippocampal gene expression

**Fig. 3:**
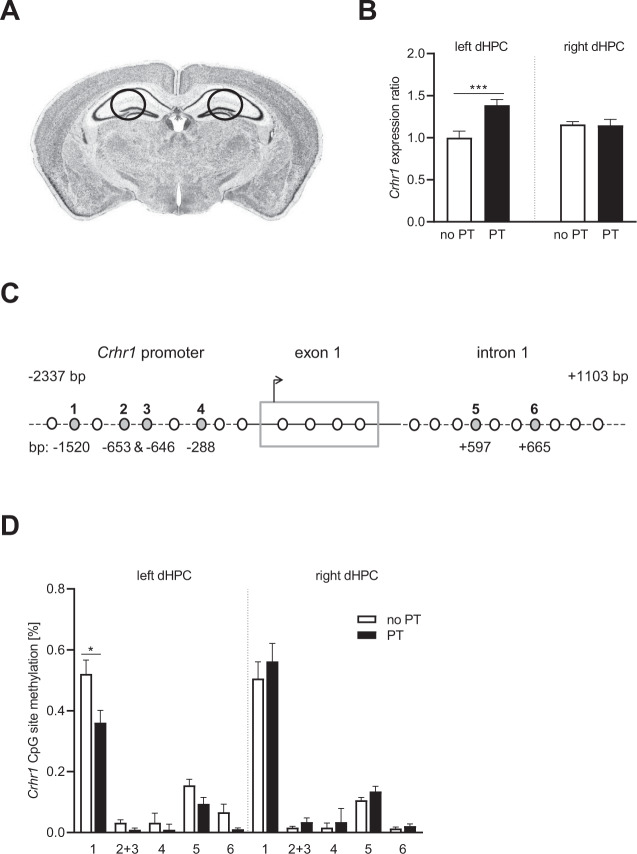
Expression and methylation analysis of *Crhr1* in the dHPC of PT adult male mice compared to a no PT control group. **A** Representative microphotograph with labels of the dissected dHPC region. **B** Results of the expression analysis of *Crhr1* in the left and right dHPC of PT adult male mice compared to a control group. The dotted gray line separates the results from left and right dHPC. *Crhr1* expression was significantly increased in the left dHPC of PT offspring (***p < 0.001, post hoc Fisher’s LSD test), but not changed in the right dHPC as compared to no PT mice. **C** Schematic representation of the *Crhr1* region of interest and localization of CpG sites (white and gray circles) investigated by methylation analysis. The promoter, the first exon and intron of *Crhr1* were analyzed for methylation changes. The gray circles indicate the position of differentially methylated CpG sites as identified by bisulfite conversion and mass spectroscopy. The shown positions of the respective CpG sites are based on NC_000077.6 (*Mus musculus* strain C57BL/6J chromosome 11, GRCm38.p2). **D** Differentially methylated *Crhr1* CpG sites in the left and right dHPC of PT and no PT mice. The dotted gray line separates the results from left and right dHPC. CpG site 2 + 3 are in juxtaposition to each other and thus have been analyzed together due to technical limitations. *p < 0.05, independent samples *t*/*u*-test. See “Results” for detailed statistical analysis.

In order to study the mechanism of HPA axis changes following prenatal trauma exposure and being raised by a traumatized mother, we measured the expression and methylation changes of the HPA axis key genes *Crhr1, Nr3c1, Nr3c2,* and *Fkbp5* in the left and right dHPC (the punched areas are outlined in Fig. 3A) of PT and no PT mice on PND 150. Analysis of *Crhr1* expression identified a significant effect of PT (*F*_1,24_ = 8.422, *p* = 0.008) and an interaction of the factors PT and hemisphere (*F*_1,24_ = 9.348, *p* = 0.005), with a significant increase of *Crhr1* mRNA levels in the left dHPC of PT offspring as compared to no PT mice (*p* < 0.001, Fisher’s LSD test) and compared to the right dHPC (*p* = 0.016, Fisher’s LSD test) (Fig. 3B). No trauma-related differences in *Crhr1* mRNA levels were detected between no PT and PT mice regarding the right dHPC (*p* = 0.914 [n.s.], Fisher’s LSD test), and *Crhr1* expression in the dHPC of no PT offspring did not differ significantly between hemispheres either (*p* = 0.098 [n.s.], Fisher’s LSD test).

**Fig. 4:**
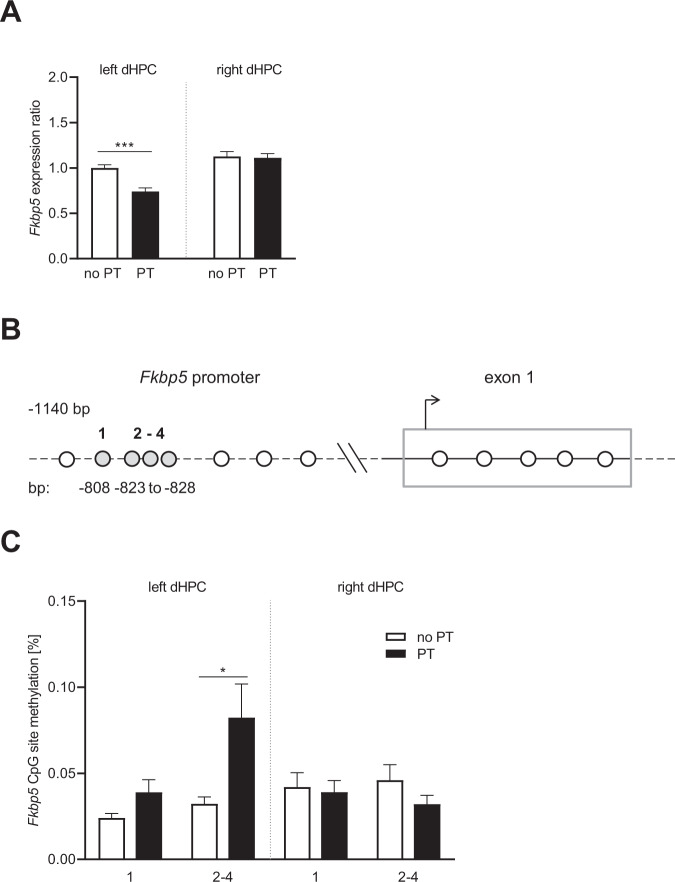
Expression and methylation analysis of *Fkbp5* in in the dHPC of PT adult male mice compared to a no PT control group. **A** Expression analysis of *Fkbp5* in the left and right dHPC (the dotted gray line separates the results from left and right dHPC). *Fkbp5* expression was significantly decreased in the left dHPC of PT offspring (***p < 0.001, post hoc Fisher’s LSD test), but not changed in the right dHPC as compared to no PT mice. **B** The promoter and the first exon of *Fkbp5* were analyzed for methylation changes. The gray circles indicate the differentially methylated CpG sites. The positions of the respective CpG sites are based on NC_000083.6 (*Mus musculus* strain C57BL/6J chromosome 17, GRCm38.p4). **C** Differentially methylated *Fkbp5* CpG sites in the left and right dHPC of PT and no PT mice. The dotted gray line separates the results from left and right dHPC. CpG sites 2–4 are in juxtaposition to each other and thus have been analyzed together due to technical limitations. *p < 0.05, independent samples *t*/*u*-test. See “Results” for detailed statistical analysis.

Additionally, two-way ANOVA revealed a significant effect of PT (*F*_1,22_ = 9.121, *p* = 0.006) and an interaction of the factors PT and hemisphere (*F*_1,22_ = 7.229, *p* = 0.013) for *Fkbp5* expression, which was significantly decreased in the left dHPC of PT mice (left dHPC, no PT vs. PT: *p* < 0.001; PT, left vs. right dHPC: *p* < 0.0001, Fisher’s LSD test) (Fig. 4A). Comparing the right dHPC of PT and no PT mice, as well as both hemispheres of the latter, *Fkbp5* expression was not significantly altered (right dHPC, no PT vs. PT: *p* = 0.817 [n.s.]; no PT, left vs. right dHPC: *p* = 0.061 [n.s.], Fisher’s LSD test) (Fig. 4A).

Regarding the expression of *Nr3c1* and *Nr3c2*, no differences were detected in the right and left dHPC (data not shown).

### PT and/or being raised by a traumatized mother is associated with long-term changes in hippocampal gene methylation

Methylation analysis of the promoter region of *Crhr1* (Fig. 3C) in the left dHPC revealed a significant hypomethylation in CpG site #1 at −1520 bp (*t* = 2.628, df = 18, *p* = 0.02) and in two CpG sites with a trend toward significance (*t* = 2.73, df = 16, *p* = 0.06; *t* = 2.09, df = 13, *p* = 0.06) in PT mice (Fig. 3D). Moreover, we identified two additional hypomethylated CpG sites in the first intron of *Crhr1* (Fig. 3C); however, differences did not reach statistical significance (*t* = 1.989, df = 13, *p* = 0.06; *t* = 1.988, df = 18, *p* = 0.06) (Fig. 3D). No methylation differences between PT and the control group were identified in the right dHPC (Fig. 3D). The analysis of the methylation status of the *Fkbp5* promoter region (Fig. 4B) revealed a significant hypermethylation of CpG sites #2–4 (*t* = 2.495, df = 16, *p* = 0.02) and a trend to hypermethylation at CpG site #1 (*t* = 1.985, df = 17, *p* = 0.06) in the left dHPC of adult PT mice (Fig. 4C). No such differences were observed at CpG sites in the right dHPC of the same animals (Fig. 4C). Also, no PT-related changes in the methylation patterns were detected for the investigated regions of *Nr3c1* and *Nr3c2* in the dHPC (data not shown).

### No expression changes of HPA axis-related genes in the fetal dHPC following PT

To examine whether or not the observed expression changes in the adult dHPC already occur *in utero*, we performed qPCR analysis in the dHPC of PT and control fetuses on GD 18, i.e., 6 days after MT procedures.

**Fig. 5:**
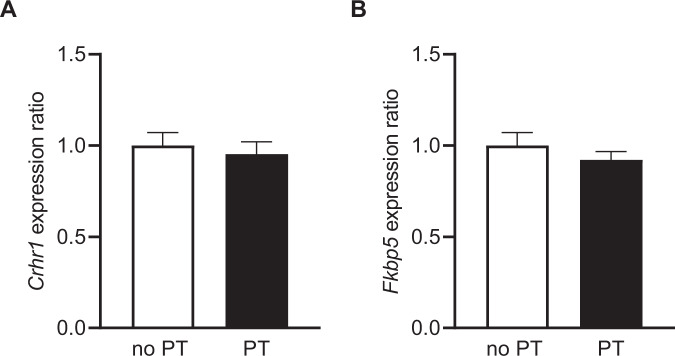
Expression analysis of *Crhr1* and *Fkbp5* in the dHPC of PT fetuses compared to a no PT control group. Expression analysis of **A** *Crhr1* and **B** *Fkbp5* in the dHPC of male fetuses on GD 18 revealed no PT-related alterations. See “Results” for detailed statistical analysis.

However, the changes in *Crhr1* and *Fkbp5* mRNA levels observed in the dHPC of adult PT and no PT mice were not detected in the dHPC of PT fetuses on GD 18 (*Crhr1*: *t* = 0.478, df = 13, *p* = 0.64 [n.s.]; *Fkbp5*: *t* = 0.950, df = 13, *p* = 0.359 [n.s.]) (Fig. 5A, B). We also did not find PT-related differences with regard to the hippocampal expression of *Nr3c1* and *Nr3c2* (data not shown).

## Discussion

In the present work, we used our previously established mouse model of prenatal trauma^17^ in order to investigate the impact of PT on the regulation of the stress system. Previously, we demonstrated that PT pups are born smaller and remain smaller throughout their life^17^ and that, when raised by traumatized mothers, PT pups display higher circulating CORT levels and increased anxiety-like behavior. Additionally, we observed high anxiety levels and poor maternal care along with reduced serum prolactin and elevated serum CORT concentrations in dams following MT^17^.

In the present study, the expression levels and methylation profiles of several key regulatory genes of the stress axis were assessed in the dHPC of PT mice in order to understand the mechanisms by which prenatal traumatic experience leads to increased CORT and anxiety levels. We confirmed our previous findings showing that maternal traumatic experience causes HPA axis dysregulation, manifesting in increased basal and stress-induced CORT levels, both in traumatized mothers and their offspring. We failed to detect a significant interaction of stress and trauma with regard to CORT levels, indicating a trauma-induced increase in CORT levels but not in CORT reactivity.

We further detected increased basal *Crhr1* mRNA levels in the left dHPC of PT mice. Notably, these changes in gene expression were accompanied by a correspondingly altered methylation pattern, namely, hypomethylated CpG sites in the *Crhr1* promoter region*. Crhr1* is widely expressed in the mammalian brain and involved in the activation of the HPA axis^23^. Interestingly, regarding the increased anxiety-like behavior of PT mice, it has been suggested that higher levels of CRHR1 induce an anxiety-like phenotype in rodents^11^. Moreover, Labermeier et al.^24^ reported on an impact of increased *Crhr1* expression on stress vulnerability in male mice^24^. Our present results are in agreement with these findings, further corroborating a potential role of *Crhr1* in the higher risk of developing anxiety disorders following prenatal trauma and/or being raised by a traumatized mother.

We were also able to detect a decrease in the expression levels of *Fkbp5* in the left dHPC, along with a significant hypermethylation of three CpG sites in the *Fkbp5* promoter region in the left dHPC of adult PT mice. Though *Fkbp5* regulation can clearly be seen from a developmental perspective as having a life-long, potentially dynamic modulation^25^, our findings were unexpected in terms of their direction since previous studies have shown that early life trauma and early life stress exposure in rodents induce *Fkbp5* demethylation and upregulation of *Fkbp5* expression in the amygdala, leading to GR-resistance and a prolonged stress response^26^.

However, *Fkbp5* methylation and expression might be subject to specific and differential regulation in individual limbic brain regions. For instance, Szymanska et al.^27^ found that prenatal trauma resulted in HPA axis hyperactivity in prenatally stressed rats, along with an increased density of GR and nonsignificantly lowered levels of FKBP51 in the hippocampus, whereas in the frontal cortex, FKBP51 levels were significantly decreased and those of GR unchanged^27^. These results were considered surprising, since the hippocampus predominantly exerts an inhibitory effect on HPA axis activity^28^, and the lack of such effect — resulting in HPA axis hyperactivity, as observed in PT rats — should rather be associated with reduced GR in prenatally stressed animals^27^. However, our findings in PT mice point in a similar direction, suggesting a PT-related hippocampal dysregulation. Interestingly, Wei et al.^29,30^ demonstrated that overexpression of GR in the forebrain of mice resulted in normal basal CORT levels, but in an increased anxiety-like behavior and a deficit in negative feedback with elevated circulating CORT after chronic stress exposure^29,30^. Considering that stress processing involves complex, integrated circuit regulation^28^, we hypothesize that PT might have a different impact on *Fkbp5* expression in the dHPC than in the amygdala and likely involves distinct epigenetic changes that need to be investigated by longitudinal studies, dissecting the effects of trauma exposure, adaptation processes, and subsequent development of the disease.

Surprisingly, we found that these methylation and expression differences were present only in the left, but not the right dHPC. However, Shipton et al.^31^ observed an effect of the left HPC on associative spatial long-term memory, which did not occur after manipulating the right HPC^31^. These findings support the idea of a lateralized long-term memory processing in mice, which, in turn, could explain why methylation differences in *Crhr1* and *Fkbp5* are only detectable in the left dHPC.

The range of these changes in DNA methylation lay between 2 and 10%. This is consistent with findings of others, supporting a model of even small changes (≤10%) in DNA methylation contributing to complex disease phenotypes^32–34^. For instance, Sudermann et al.^35^ identified a significant impact of early life stress on *Nr3c1* gene expression and HPA axis responsiveness in rats, which was based on small methylation changes (2–4%) in the promoter region of *Nr3c1*^35^. In contrast to these findings and those of Müller et al.^36^, who reported on *Crhr1*-dependent *Nr3c2* expression in the dHPC of mice^36^, we could not detect a significant effect of PT on the expression and methylation of *Nr3c1* or *Nr3c2*, although *Crhr1* expression was altered. However, this might be explained by the different experimental setting of our study, since we analyzed basal gene expression, whereas Müller et al.^36^ focused on stress-induced changes.

Exposure of the pups to increased maternal CORT concentrations through the blood has been discussed as a possible mechanism for effects of PT. Here, we investigated whether the observed alterations in gene expression can already be found prenatally (i.e., shortly after trauma and independently of the postnatal environment). Since the changes in *Fkbp5* and C*rhr1* mRNA levels in the dHPC were not detected at GD 18, they are likely to occur after birth. However, we are aware that the validity of this conclusion is limited, as due to technical reasons, fetal gene expression in the dHPC was analyzed without distinction between hemispheres. We aim to clarify this issue in future studies and to investigate whether prenatally unaltered expression of the respective genes is due to a delayed manifestation or to being raised by a traumatized mother. Moreover, future work should include the examination of protein levels to complete and support our results obtained by gene expression and methylation analyses, which we focused on in the present work due to a limited number of PT/no PT offspring and thus availability of dHPC samples.

In summary, prenatal trauma is known to have an impact on HPA axis functioning, and accordingly, our study revealed increased *Crhr1* and decreased *Fkbp5* expression in the left dHPC of PT mice. These differences in gene expression were accompanied by corresponding hypo- and hypermethylation of CpG sites in the promoter regions of *Crhr1* and *Fkbp5*, respectively. Moreover, we detected an increased basal and post stress CORT secretion in these animals. Our findings provide evidence that prenatal trauma and/or being raised by a traumatized mother has a long-term epigenetic impact on stress axis function and anxiety phenotype of the offspring.
